# The plasmid-encoded members of paralogous gene family 52 are dispensable to the enzootic cycle of *Borrelia burgdorferi*

**DOI:** 10.1128/iai.00214-24

**Published:** 2024-08-09

**Authors:** Ashley M. Groshong, Nora E. Gibbons, Brendan P. Moore, William T. Bellamy, Jon S. Blevins

**Affiliations:** 1 Department of Microbiology and Immunology, University of Arkansas for Medical Sciences, Little Rock, Arkansas, USA; 2 Department of Medicine, UConn Health, Farmington, Connecticut, USA; 3 Laboratory of Bacteriology, Rocky Mountain Laboratories, Division of Intramural Research, National Institute of Allergy and Infectious Diseases, National Institutes of Health, Hamilton, Montana, USA; 4 Department of Pathology, University of Arkansas for Medical Sciences, Little Rock, Arkansas, USA; Washington State University, Pullman, Washington, USA

**Keywords:** Lyme disease, *Borrelia*, *Borrelia burgdorferi*, borreliella, molecular genetics, pathogenesis, spirochetes, vector-borne diseases, tick-borne pathogens

## Abstract

Lyme disease, the leading vector-borne disease in the United States and Europe, develops after infection with *Borrelia burgdorferi sensu lato* bacteria. Transmission of the spirochete from the tick vector to a vertebrate host requires global changes in gene expression that are controlled, in part, by the Rrp2/RpoN/RpoS alternative sigma factor cascade. Transcriptional studies defining the *B. burgdorferi* RpoS regulon have suggested that RpoS activates the transcription of paralogous family 52 (PFam52) genes. In strain B31, PFam52 genes (*bbi42*, *bbk53*, and *bbq03*) encode a set of conserved hypothetical proteins with >89% amino acid identity that are predicted to be surface-localized. Extensive homology among members of paralogous families complicates studies of protein contributions to pathogenicity as the potential for functional redundancy will obfuscate findings. Using a sequential mutagenesis approach, we generated clones expressing a single PFam52 paralog, as well as a strain deficient in all three. The single paralog expressing strains were used to confirm BBI42, BBK53, and BBQ03 surface localization and RpoS regulation. Surprisingly, the PFam52-deficient strain was able to infect mice and complete the enzootic cycle similar to the wild-type parental strain. Indeed, the presence of numerous pseudogenes that contain frameshifts or internal stop codons among the PFam52 genes suggests that they may be subjected to gene loss in *B. burgdorferi*’s reduced genome. Alternatively, the lack of phenotype might reflect the limitations of the experimental mouse infection model.

## INTRODUCTION

*Borrelia burgdorferi* is a zoonotic pathogen, which must transit between its tick vector, *Ixodes scapularis*, and a vertebrate host (1–3). Effective colonization of both the mammalian host and the tick vector is facilitated by an extensive shift in the production of surface lipoproteins that are important for migration, adhesion, and immune evasion (4–7). Many environmental stimuli (e.g., pH, temperature, cell density, and growth rate) coordinate to trigger this change in gene regulation for spirochetes residing within the tick midgut (8–14). These regulatory changes serve as cues for spirochete migration from the tick midgut, into the salivary glands, and subsequent deposition into the host at the site of tick attachment.

To accurately synchronize gene regulatory events with the enzootic cycle, *B. burgdorferi* utilizes two complex regulatory systems that globally control gene expression in the vector or host environments (15–17). Genes important for survival in the tick are primarily modulated by the two-component system Hk1/Rrp1 which generates cyclic-di-GMP, a dinucleotide second messenger (18–20). As the tick takes a bloodmeal and spirochetes begin the transit to the mammal, a second two-component system, Hk2/Rrp2, activates an alternative sigma factor cascade, RpoN/RpoS, resulting in differential expression of genes necessary to aid the spirochete in mammalian colonization (21–24). In addition, a number of transcriptional modulators have been shown to influence RpoS transcription (BosR, CsrA, DsrA), though RpoS is considered a “gatekeeper” to spirochete transmission and mammalian infection (21).

The *B. burgdorferi* genome contains numerous paralogous gene families (PFams), many of which are surface-localized (25). Analysis of multiple *B. burgdorferi* strains has identified 160 PFams, most of which contain several paralogs; the potential for functional redundancy can complicate the characterization of the individual genes’ contribution to the infectious cycle *via* a mutagenesis approach. PFams 47 (Dbps), 54 (CRASP1/BBA68), 163 (CRASP3/5), 36 (Bmps), and 37 (OppAs) have been previously studied in *B. burgdorferi* (26–51). However, accurate analysis of their functional contribution to infection and the enzootic cycle is complicated by the potential for compensatory redundancy: (i) DbpA/B are both required for normal murine infection, (ii) CspA (CRASP-1/BBA68) is important for spirochete survival in fed nymphal ticks and transmission to mammalian hosts, BBA64 and BBA66 are required for mammalian infection *via* tick transmission, but the contributions of the other PFam54 members have not been reported, (iii) CRASP3/5 provides complement resistance to *B. burgdorferi*, but their contributions to murine infection are unknown, (iv) BmpA/B mutants can infect mice but fail to induce severe arthritis, and the roles of BmpC/D are unknown, and (v) OppA1 is critical for tick survival and OppA2 for mammalian dissemination, OppA4 and OppA5 are not essential for mammalian infection, OppA5 for persistence in the mammal, and OppA3 has yet to be evaluated. PFam52 is a family of putatively RpoS-regulated outer surface lipoproteins that contain only three paralogs in strain B31. Previous studies have identified PFam52 members, *bbi42*, *bbk53*, and *bbq03* as differentially regulated in relation to *rpoS* expression by transcriptional microarray and RNAseq (8, 21, 52–54). In addition, protein microarray studies have shown BBQ03 and BBI42 as prominent late-stage Lyme disease antigens in humans (55). In this study, we utilized strains expressing single PFam52 paralogs to confirm their regulation and localization; all three paralogs are positively regulated by RpoS and surface-localized. We confirmed their role as readily detectable antigens at late stages of infection; however, we were surprised to find that a PFam52-deficient mutant was not only able to infect mice at wild-type levels but was also able to effectively complete the enzootic cycle. As such, the contribution of PFam52 surface proteins to the lifecycle of *B. burgdorferi* still remains undefined.

## RESULTS

### Comparison of PFam52 members in Lyme *Borrelia*

**Fig 1:**
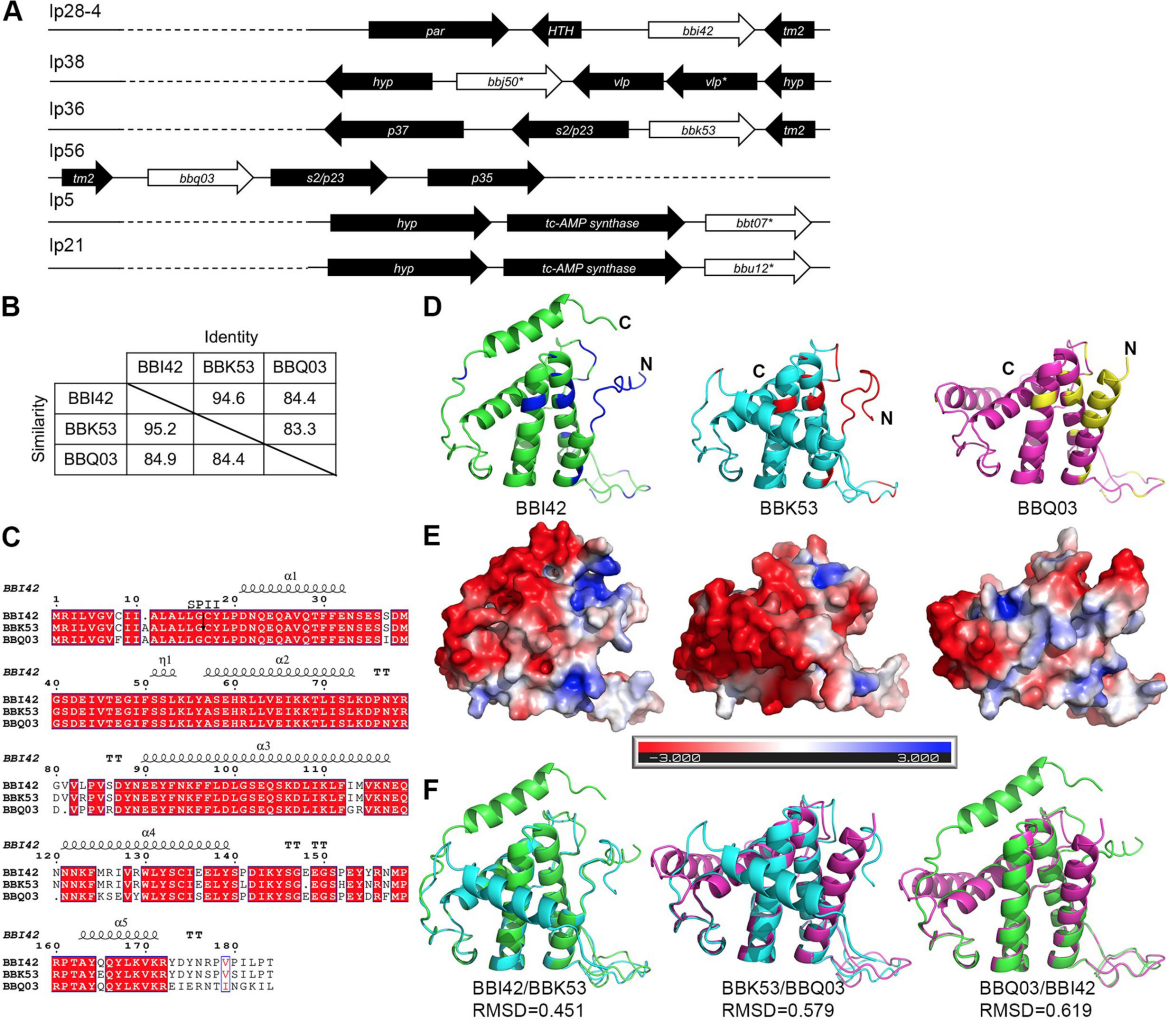
(**A**) Gene arrangement of PFam52 paralogs on plasmids. Asterisks denote pseudogenes with frameshift or stop codons. (**B**) Identity and similarity index for PFam52 paralogs as determined using MUSCLE. (**C**) Alignment of PFam52 paralogs where red highlight denotes identity and blue box denotes similarity. SPII indicates the signal peptidase II cleavage site. (**D**) AlphaFold models of BBI42 (green), BBK53 (teal), and BBQ03 (magenta) with variable amino acids highlighted in blue, red, and yellow, respectively. (**E**) Electrostatics distribution over protein surface on a scale of +3 to −3. (**F**) Alignments of BBI42, BBK53, and BBQ03 models.

The genome of strain B31 contains six PFam52 members; however, *bbi42, bbk53,* and *bbq03* are the only predicted ORFs that do not contain premature stop codons, frameshifts, or truncations. PFam52 members are limited to Lyme *Borrelia*, with most strains containing multiple gene paralogs along with pseudogenes. Phylogenetically, the paralogs from the Old World and New World strains cluster separately, as expected (Fig. S1 and S2). *B. burgdorferi* strain JD1 contains paralogs to all the B31 genes, as well as an additional BBE04 PFam52 member that appears to be unique to the Lyme disease *Borrelia*. The genome of a *Borrelia mayonii* isolate from Minnesota only encodes a single paralog similar to BBQ03. Old World strain *Borrelia afzelii* contains four more distantly related paralogs that cluster together. Interestingly, *Borrelia garinii* only encodes remnants of PFam52 with three frameshifted pseudogenes. In *B. burgdorferi* strain B31, all three genes are located on linear plasmids (Fig. 1A). *bbq03* resides on the left arm of lp56 with the promoter proximal to the telomere while *bbi42* and *bbk53* reside near the end of the right arm of lp28-4 and lp36, respectively, with promoters distal to the telomere of each plasmid. Previous studies using mutants lacking each of these plasmids individually have shown modest attenuation phenotypes in the mammal or ticks (56–59). Additional pseudogenes are present on other linear plasmids near the telomere of the right arm (*bbj50*, lp38; *bbu12*, lp21; *bbt07*, lp5; Fig. 1A); frameshifts or internal stops disrupt these genes, and they retain little similarity to the full paralogs. Each of the *bbi42, bbk53,* and *bbq03* paralogs is a predicted surface lipoprotein and shares 84.4%–95.2% similarity (Fig. 1B). Alignment of amino acid sequences demonstrates the largest variation at the C-terminal end with BBQ03 showing the greatest variation (Fig. 1C). AlphaFold models of the three proteins result in highly similar overall structures with five conserved alpha helices and disordered loops, where the N-terminal α1 position varies in each model suggesting flexibility (Fig. 1D). The variation in amino acid sequence map to both alpha helix and loop structures, with the C-terminal region of BBQ03 forming an extended alpha helix in contrast to the C-terminal disordered region of BBI42 and BBK53. When electrostatics are mapped over the predicted structures, there are differences seen in the charges within the C-terminal region that may suggest differences in ligand interactions (Fig. 1E). Alignments of these models display high RMSD values (0.451–0.619Å) with the primary amino acid variations present in the N-terminal helix, the loops, and the C-terminal end (Fig. 1F). Given the high degree of amino acid similarity and chance for functional redundancy, we deemed it necessary to create a clone deficient in all three gene products to further characterize their contribution to the enzootic cycle.

### Inactivation of PFam52 genes in *B. burgdorferi*

**Fig 2:**
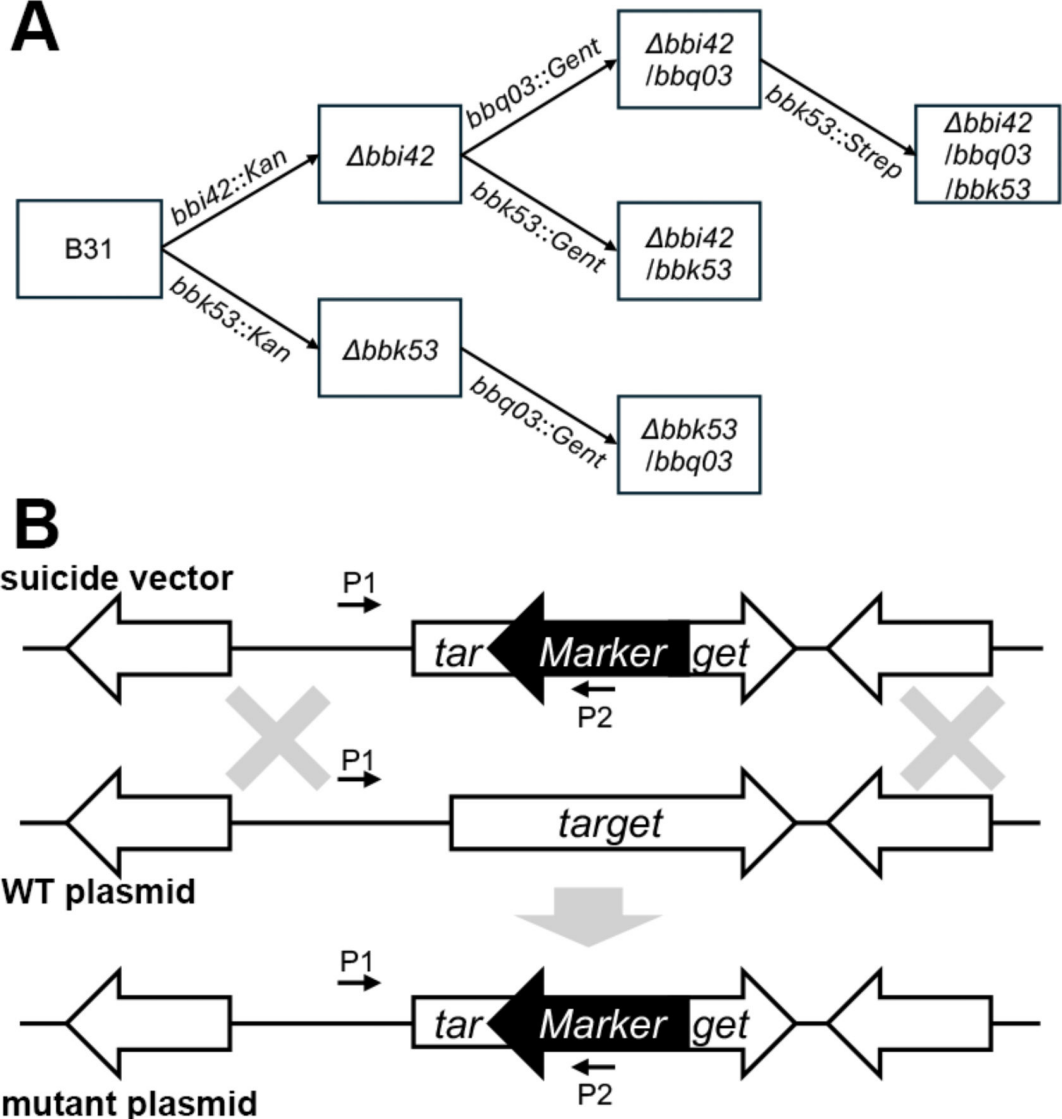
Generation of strains expressing a single PFam52 paralog and a PFam52 null strain. (**A**) Schematic of stepwise mutagenesis approach. Generated strains are represented in boxes and the mutagenesis constructs are noted on arrows. (**B**) Diagram illustrating the homologous recombination between the target gene (*bbi42*, *bbk53*, or *bbq03*) and the suicide vector. Small arrows designate relative positions of diagnostic primers.

Due to the high amino acid identity of the PFam52 paralogs, it was necessary to produce strains expressing only one paralog to evaluate expression and localization. In addition, a triple mutant would be required to assess the functional role of the PFam52 paralogs throughout the enzootic cycle. A sequential mutagenesis approach was developed utilizing antibiotic markers Kan^r^, Gent^r^, and Strep^r^ (Fig. 2A). Mutagenesis constructs were generated by amplifying upstream- and downstream-flanking regions of the targeted gene from strain B31 and a Kan^r^, Gent^r^, or Strep^r^ marker was ligated between the two flanking regions, replacing an internal region of the open reading frame (Fig. 2B). Due to sequence similarity among target genes, PCR screening for antibiotic insertions was performed with a primer unique to the upstream region of the gene target and a primer in the antibiotic cassette (P1 and P2; Fig. 2B).

To generate a strain capable of producing only BBI42, strain B31 was first electroporated with pJSB532 (*bbk53::Kan*) and transformants were recovered and screened by PCR for Kan^r^ insertion. A single clone (*Δbbk53*) positive for *bbk53::Kan* and confirmed to contain all B31 endogenous plasmids was selected for further modification. Clone *Δbbk53* was subsequently electroporated with pJSB566 (*bbq03::Gent*) and transformants were recovered and screened by PCR for Gent^r^ insertion. A single clone (*Δbbk53Δbbq03*) positive for *bbq03::Gent* and confirmed to match the parent B31 plasmid profile was selected for further characterization as a strain able to produce only BBI42.

To generate a strain capable of producing only BBQ03, B31 was first electroporated with pJSB528 (*bbi42::Kan*), and transformants were recovered and screened by PCR for Kan^r^ insertion. A single clone (*Δbbi42*) positive for *bbi42::Kan* and confirmed to contain all B31 endogenous plasmids was selected for further modification. Clone *Δbbi42* was subsequently electroporated with pJSB569 (*bbk53::Gent*), and transformants were recovered and screened by PCR for Gent^r^ insertion. A single clone (*Δbbi42Δbbk53*) positive for *bbk53::Gent* and confirmed to match the parent B31 plasmid profile was selected for further characterization as a strain able to produce only BBQ03.

To generate a strain that only produces BBK53, clone *Δbbi42* was electroporated with pJSB566 (*bbq03::Gent*), and transformants were recovered and screened by PCR for a Gent^r^ insertion. A single clone (*Δbbi42Δbbq03*) positive for *bbq03::Gent* and confirmed to contain all B31 endogenous plasmids was selected for further characterization as a strain able to produce only BBK53. Finally, to create the triple mutant, strain *Δbbi42Δbbq03* was electroporated with pJSB592 (*bbk53::Strep*), and transformants were recovered and screened by PCR for Strep^r^ insertion. A single clone (*Δbbi42Δbbk53Δbbq03*) positive for *bbk53::Strep* and confirmed to match the parent B31 plasmid profile (Fig. S3) was selected for further characterization as a PFam52-deficient strain.

### PFam52 genes are expressed *in vitro*

**Fig 3:**
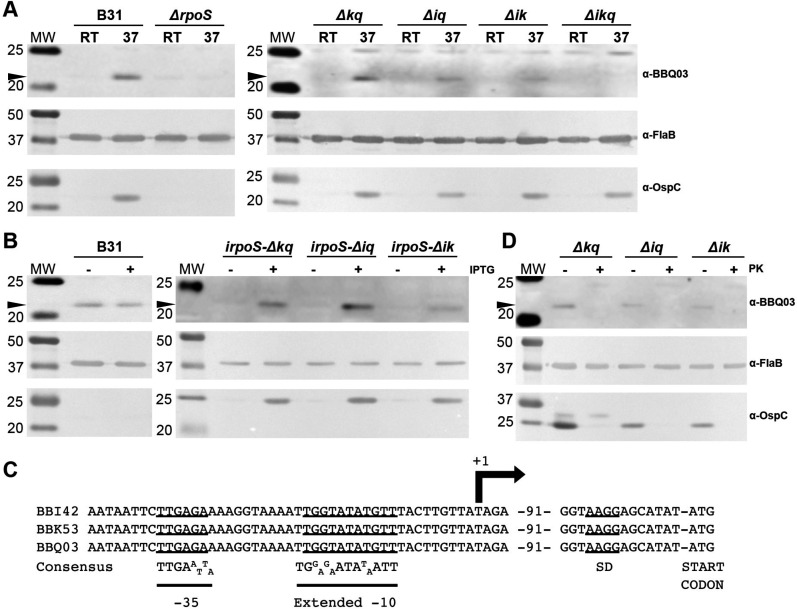
(**A-B**) Immunoblot analysis of PFam52 expression. (**A**) B31, *ΔrpoS,* and *Δbbk53Δbbq03* (*Δkq*), *Δbbi42Δbbk53* (*Δik*), *Δbbi42Δbbq03* (*Δiq*), and *Δbbi42Δbbk53Δbbq03* (*Δikq*) at low density, room-temperature/pH 7.5 (RT) or high density, 37°C/pH 6.8 (37). (**B**) IPTG induction of B31 *irpoS-Δbbk53Δbbq03* (*irpoS-Δkq*), *irpoS-Δbbi42Δbbk53* (*irpoS-Δik*), and *irpoS-Δbbi42Δbbq03* (*irpoS-Δiq*). (**C**) TSS and promoter map for PFam52 paralogs. (**D**) Proteinase K accessibility assay for *Δbbk53Δbbq03* (*Δkq*), *Δbbi42Δbbk53* (*Δik*), *Δbbi42Δbbq03* (*Δiq*), and *Δbbi42Δbbk53Δbbq03* (*Δikq*). All molecular weight standards are shown in kDa (MW). The arrowheads on the left denote the relative positions of the Pfam52 paralogs in the immunoblots.

Previous studies indicated that PFam52 paralogs are regulated *in vitro* by increased temperature and/or RpoS activation (13, 60). Therefore, to confirm the expression of each paralog*,* wild-type, single paralog expressing, and PFam52-deficient strains were cultured at room temperature/pH 7.5 or at 37°C/pH 6.8. As controls, a wild type and a B31 strain deficient in *rpoS* (*ΔrpoS*) were included in analyses. Samples from both growth conditions were collected for immunoblot to assess BBI42, BBK53, and BBQ03 protein expression (Fig. 3A). Sera generated against recombinant BBQ03 were utilized for protein detection, as it was cross-reactive to all three PFam52 paralogs (Fig. S4A). Strain B31 expressed PFam52 paralogs when grown at 37°C/pH 6.8, conditions known to induce RpoS expression (13, 60, 61). We were able to detect a protein band for each of the single paralog-expressing strains only at 37°C/pH 6.8, confirming all three proteins are individually expressed and under conditions known to induce RpoS expression (Fig. 3A). OspC, a RpoS-regulated gene whose expression is indicative of RpoS production (62), was also upregulated under these conditions. Consistent with RpoS-dependent regulation, neither the PFam52 paralogs nor OspC was expressed in the *ΔrpoS* strain. As expected, the triple mutant produced no detectable protein, confirming no other paralogs are expressed in strain B31.

### PFam52 genes are RpoS regulated

While the above analysis suggests that the PFam52 paralogs are RpoS regulated based on temperature shifts, RpoS itself is regulated by temperature (11), making it difficult to determine if these genes are temperature-regulated or RpoS-regulated. In addition, some of the PFam52 paralogs have been identified in transcriptional studies as being positively regulated by RpoS (8, 52–54, 63); however, *in vitro* culture conditions that initiate *rpoS* transcription (high density, 37°C, pH 6.8) can also activate other factors that have been shown to influence *rpoS* transcription as well their putative regulons [i.e., CsrA (64) and BosR (65)]. Therefore, to deconvolute these regulatory events, we utilized strains carrying a copy of *rpoS* with *irpoS*. These strains, *irpoS-Δbbk53Δbbq03*, *irpoS-Δbbi42Δbbq03*, and *irpoS-Δbbi42Δbbk53*, allow controlled expression of *rpoS* by adding IPTG to the culture, bypassing upstream factors that produce a significant amount of regulatory crosstalk. To confirm that the addition of IPTG alone did not elicit any changes in gene regulation, strain B31 was included in all analyses. Strains were cultured at pH 7.5/37°C, then induced with IPTG during early logarithmic growth to initiate *rpoS* transcription, a timepoint during which endogenous RpoS is not robustly expressed. Samples were collected at 12 h post-induction for immunoblot analyses (Fig. 3B). Strain B31 demonstrated no change in OspC or PFam52 paralog production when grown with or without IPTG, confirming that IPTG in the absence of *irpoS* does not affect regulation. Meanwhile, the addition of IPTG to single PFam52 paralog expressing strains with *irpoS*, BBI42, BBK53, and BBQ03 was produced only when *rpoS* was induced, though BBQ03 was present at lower levels than BBI42 and BBK53 (Fig. 3B). OspC induction was used as a surrogate to confirm RpoS production, and OspC was only detected in *irpoS* strains induced with IPTG. The OspC levels on the blots are also likely lighter because we used a lower concentration of IPTG (e.g., 0.1 mM) since over-production of RpoS impairs *B. burgdorferi* growth (data not shown).

To identify the putative promoter regions of each paralog, 5′ RACE was conducted on RNA from *Δbbk53Δbbq03* (*bbi42*), *Δbbi42Δbbq03* (*bbk53*), and *Δbbi42Δbbk53* (*bbq03*) to identify the transcriptional start site (TSS) and allow alignment of the paralog promoter regions for comparison (Fig. 3C). The results of the 5′ RACE identified a common nucleotide 109 nucleotides upstream of the start codon as the TSS for all three genes. Comparison of the regions upstream of each of TSS for all three of the paralogs identified appropriately spaced RpoS-dependent promoters that are consistent with the consensus sequence previously described by Caimano et al. (52) (Fig. 3C).

### PFam52 paralogs are readily detectable surface antigens during chronic infection

A previous study demonstrated *via* transcriptional microarray that *bbi42* was highly expressed in strain 297 cultivated in dialysis membrane chambers (DMCs) in rats (52). In addition, BBI42 was identified as a prominent late-stage Lyme disease antigen in humans (55). Therefore, we evaluated antibody response to the individual PFam52 paralogs during murine infection. Recombinant BBI42, BBK53, and BBQ03 were generated in *E. coli* and purified, allowing the assessment of serological responses by immunoblot. Equal loading of recombinant protein was determined using Sypro stain, and protein was blotted with B31-infected mouse sera from 2-, 6-, and 10 weeks post-infection to evaluate the presence of PFam52 antibodies. Quantification by densitometry of reactive sera was standardized to recombinant OspC, a prominent early-stage antigen during mammalian infection (66). Antibodies against PFam52 paralogs were detectable at low levels at 2 weeks, with a dramatic increase at 6 and 10 weeks post-infection (Fig. S4B). Interestingly, at 2 weeks post-infection, infected sera demonstrated slightly different affinities to the three PFam52 paralogs, perhaps suggesting changes in antibody specificity during early infection. The PFam52 paralogs demonstrated more seroreactivity compared to OspC only during later stages of infection, correlating with previous results that identified BBI42 as a late-stage antigen.

The PFam52 paralogs are predicted to be surface lipoproteins according to LipoP, a lipoprotein prediction software (67), encoding a putative signal sequence with a signal peptidase II cleavage site between amino acids 16 and 17 for BBI42 and between amino acids 17 and 18 for BBK53 and BBQ03 (Fig. 1C). To confirm that all three PFam52 paralogs are surface localized in strain B31, strains *Δbbk53Δbbq03* (*bbi42*), *Δbbi42Δbbq03* (*bbk53*), and *Δbbi42Δbbk53* (*bbq03*) were used for proteinase K accessibility assays (Fig. 3D). Strains were cultured at 37°C/pH 6.8 to ensure paralog expression during localization assays and treated with proteinase K to cleave surface proteins. When intact cells were treated with proteinase K for 1 h, OspC, an outer surface protein control (68), was readily degraded, while FlaB, a periplasmic protein, was not degraded due to its subsurface localization. BBI42, BBK53, and BBQ03 were also degraded, demonstrating that BBI42, BBK53, and BBQ03 are located on the outer surface of the spirochete.

### PFam52 members are not required during acute or chronic murine infection

**Fig 4:**
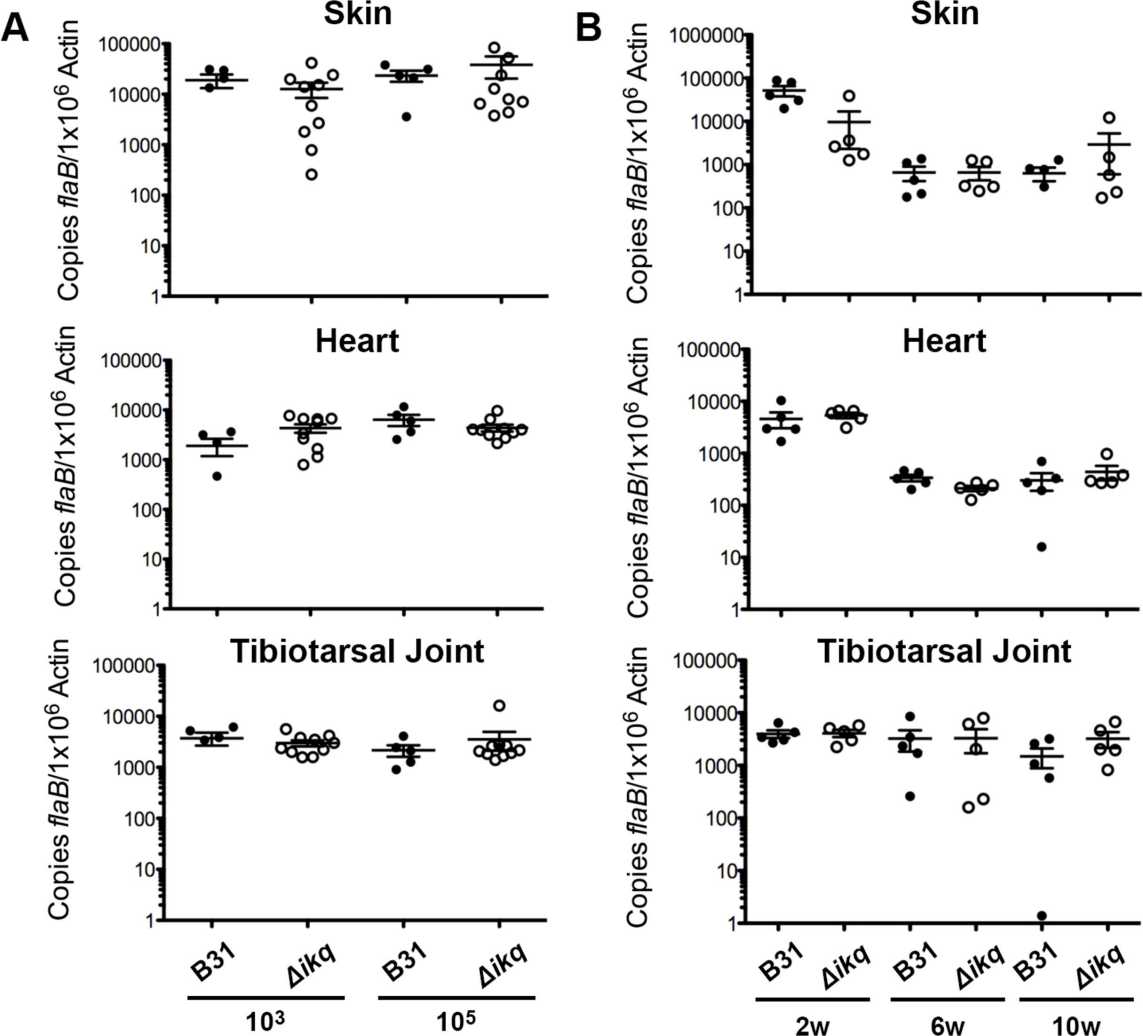
qPCR demonstrating bacterial burdens in mice infected with B31 or *Δbbi42Δbbk53Δbbq03* (*Δikq*). (**A**) Skin, heart, and tibiotarsal joints from mice inoculated at 10^3^ or 10^5^ spirochetes at 2 weeks post-infection. (**B**) Skin, heart, and tibiotarsal joints from mice infected with 10^4^ spirochetes at 2, 6, or 10 weeks post-infection. Error bars represent SEM and differences between wild type and mutant were not statistically significant.

Previous studies have determined that a *rpoS*-deficient mutant is unable to infect mice when administered by needle inoculation (69). Because RpoS acts as a global regulator to initiate the expression of genes required for bacterial adaptation to the host environment, ablation of *rpoS* expression results in spirochetes that are unable to adapt and survive in the host. Individual genes in the RpoS regulon [i.e., OspC (70)] are also essential for mammalian infection. Therefore, we sought to assess the contribution of RpoS-regulated PFam52 members during murine infection *via* needle inoculation. Groups of five C3H/HeN mice were inoculated intradermally with increasing doses (10^2^, 10^3^, 10^4^, and 10^5^ spirochetes) of parental strain B31 and two independently generated PFam52-deficient clones (*Δbbi42Δbbk53Δbbq03*). At 2 weeks post-infection, ear punch, skin (distal from injection site), lymph nodes, heart, bladder, and tibiotarsal joint were collected for culture. Tissues from both wild-type and mutant-infected mice consistently cultured positive at inoculations of 10^3^ spirochetes or higher (Table 1). The ID_50_ of the B31 parental strain was 347 spirochetes (95% CI, 120–1,023 spirochetes) and the combined ID_50_ of the two *Δbbi42Δbbk53Δbbq03* clones was 178 spirochetes (95% CI, 95–323 spirochetes), which is not a statistically significant difference. In addition, there were no tissue-specific defects in infection for the *Δbbi42Δbbk53Δbbq03* clones, suggesting the PFam52-deficient strain did not demonstrate any defects in dissemination or tissue tropism in the host. To evaluate bacterial burdens in the mutant, skin, heart, and tibiotarsal tissues were collected from mice infected with 10^3^ and 10^5^ spirochetes 2 weeks post-infection and spirochete burdens were assessed by qPCR (Fig. 4A). Bacterial burdens were similar between the two strains at both a low-dose (10^3^) and a high-dose (10^5^) inoculum, suggesting the PFam52-deficient strain demonstrates no defects in host colonization. Finally, heart and knee tissues were collected at 2 weeks post-infection at a dose of 1 × 10^3^ for blinded histopathological assessment (Fig. S5). There was no statistically significant difference between histology scores for strain B31 and *Δbbi42Δbbk53Δbbq03* in either the heart or knee tissues.

**TABLE 1:** ID_50_ tissue culture of *Δbbi42Δbbq03Δbbk53* 2 weeks post-infection

Strain/dose	No. of positive cultures/total							No. infected mice/total	ID_50_
	Ear punch	Back skin	Lymph nodes^*a*^	Heart	Tibiotarsal joint	Bladder	All sites		
B31									
10^2^ 10^3^ 10^4^ 10^5^	1/5 4/5 5/5 5/5	1/5 2/5 4/5 5/5	1/5 4/5 5/5 5/5	1/5 4/5 5/5 5/5	1/5 4/5 5/5 5/5	1/5 4/5 5/5 5/5	6/30 22/30 29/30 30/30	1/5 4/5 5/5 5/5	347
*Δbbi42Δbbq03Δbbk53* clone 1									
10^2^ 10^3^ 10^4^ 10^5^	2/5 4/5 5/5 4/5	3/5 5/5 4/5 5/5	3/5 5/5 5/5 5/5	3/5 5/5 5/5 5/5	3/5 5/5 5/5 5/5	3/5 5/5 5/5 5/5	17/30 29/30 29/30 29/30	3/5 5/5 5/5 5/5	<105
*Δbbi42Δbbq03Δbbk53* clone 2									
10^2^ 10^3^ 10^4^ 10^5^	0/5 5/5 5/5 4/5	0/5 5/5 5/5 5/5	0/5 5/5 5/5 5/5	0/5 5/5 5/5 5/5	0/5 5/5 5/5 5/5	0/5 4/5 5/5 5/5	0/30 29/30 30/30 29/30	0/5 5/5 5/5 5/5	437

^*a*^ Node pairs were collected and cultured separately, all node pairs cultured consistently and are summarized here.

While infection studies demonstrated no defects in host colonization and dissemination at 2 weeks post-infection for the mutant, the observation that BBQ03 is a late-stage, readily detectable antigen during *B. burgdorferi* infections suggested a possible role during chronic infection. To assess the longitudinal infection phenotype, groups of five C3H/HeN mice were intradermally injected *via* needle inoculation of 10^4^ spirochetes of B31 or *Δbbi42Δbbk53Δbbq03*. At 2, 6, and, 10 weeks post-infection, skin, heart, and tibiotarsal tissues were collected and the presence of spirochetes was determined by tissue culture (Table 2). There was no difference between the parental strain and *Δbbi42Δbbk53Δbbq03* as assessed by tissue culture. qPCR analysis was also conducted on skin, heart, and tibiotarsal tissue samples at 2, 6, and 10 weeks post-infection, which demonstrated no difference in spirochete burdens between the two strains at any timepoint evaluated (Fig. 4B).

**TABLE 2:** Longitudinal tissue culture of *Δbbi42Δbbq03Δbbk53*

Strain/duration	No. of positive cultures/total							No. infected mice/total
	Ear punch	Back skin	Lymph nodes^*a*^	Heart	Tibiotarsal joint	Bladder	All sites	
B31								
2-week PI 6-week PI 10-week PI	5/5 5/5 3/5	5/5 4/5 4/5	5/5 5/5 4/5	5/5 5/5 4/5	5/5 4/5 3/5	5/5 5/5 4/5	30/30 28/30 22/30	5/5 5/5 4/5
*Δbbi42Δbbq03Δbbk53*								
2-week PI 6-week PI 10-week PI	4/5 5/5 4/5	5/5 5/5 5/5	5/5 5/5 5/5	5/5 5/5 5/5	5/5 4/5 5/5	5/5 5/5 5/5	29/30 29/30 29/30	5/5 5/5 5/5

^*a*^ Node pairs were collected and cultured separately, all node pairs cultured consistently and are summarized here.

### PFam52 members are not essential for the maintenance of the enzootic cycle

**Fig 5:**
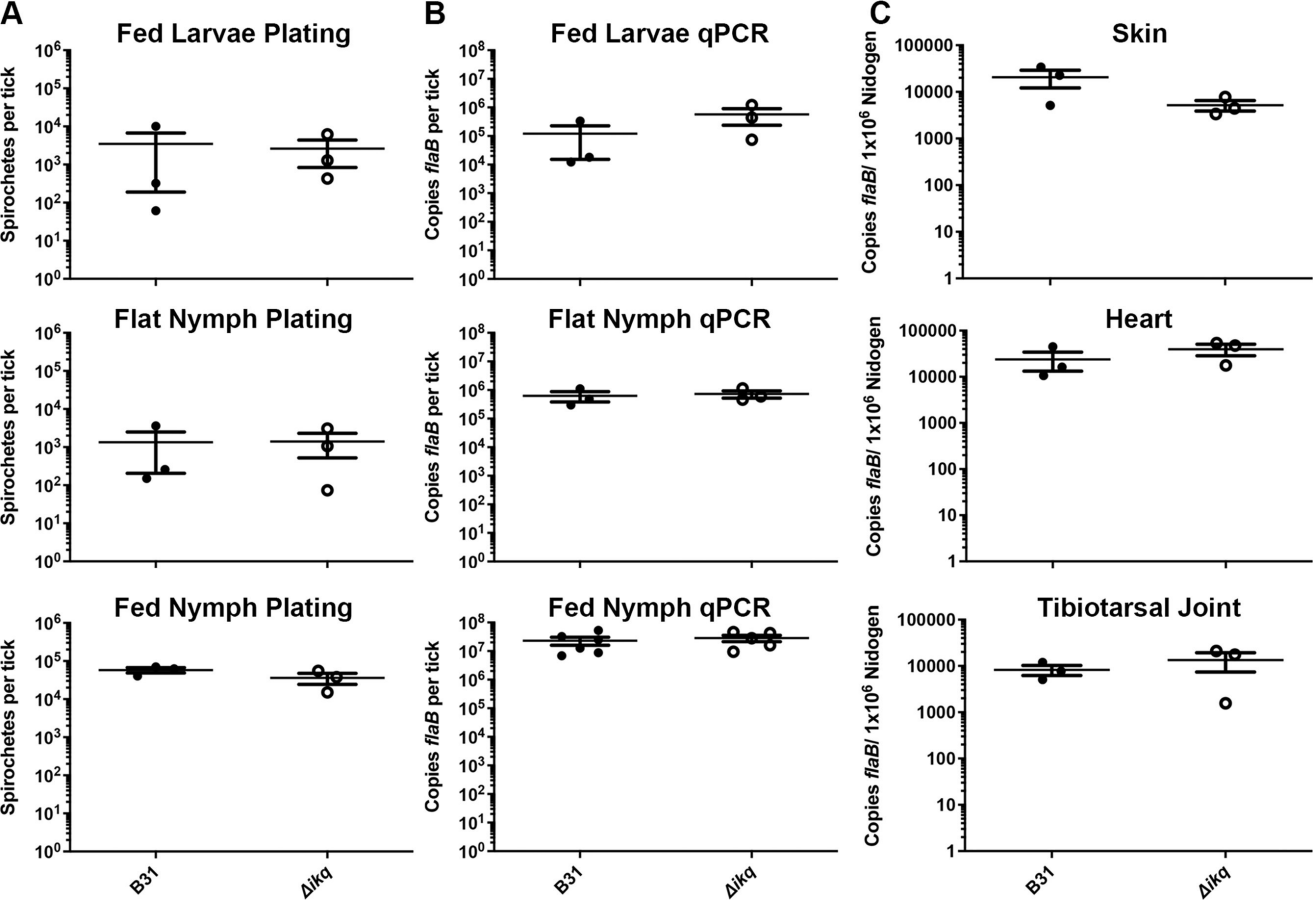
(**A-B**) Analysis of pooled fed larvae (drop-off), flat nymphs (post-molt), and fed nymphs (drop-off) burdens by (**A**) semisolid plating or (**B**) qPCR for B31 or *Δbbi42Δbbk53Δbbq03* (*Δikq*). (**C**) Skin, heart, and tibiotarsal joints from mice inoculated by nymphs colonized with B31 or *Δbbi42Δbbk53Δbbq03* (*Δikq*) at 4 weeks post-infection. Error bars represent SEM and differences between wild type and mutant were not statistically significant.

While RpoS regulation suggests that the PFam52 members may be important for host infection, some RpoS-regulated genes are essential for transmission from tick to mammal (71). To determine whether the PFam52 members play a role in the tick vector, we compared the ability of *Δbbi42Δbbk53Δbbq03* and the parental strain to complete the enzootic cycle. Needle-inoculated mice were parasitized by naïve larvae to generate naturally infected larvae. Larval burdens were assessed at post-repletion and nymphal burdens post-molt *via* viability plating and qPCR (Fed Larvae and Flat Nymphs, respectively; Fig. 5A and B). *Δbbi42Δbbk53Δbbq03* demonstrated similar burdens to the parental strain at both stages. Infected nymphs were subsequently allowed to parasitize naïve mice until repletion. Nymphal burdens also showed no difference between wild type and mutant by viability plating and qPCR (Fig. 5A and B). Nymph-infected mice were evaluated for infection 2 weeks post-infestation by tissue culture and qPCR (Table 3; Fig. 5C). There were no differences found between the parental strain and *Δbbi42Δbbk53Δbbq03* either in tissue culture or burdens, confirming both transmission and colonization. These data suggest that the PFam52 members are dispensable for maintaining the *B. burgdorferi* infectious cycle.

**TABLE 3:** Nymph-infected tissue culture of *Δbbi42Δbbq03Δbbk53* four weeks post-infestation

Strain	No. of positive cultures/total							No. infected mice/total
	Ear punch	Back skin	Lymph nodes^*a*^	Heart	Tibiotarsal joint	Bladder	All sites	
B31								
4-week PI	3/3	3/3	3/3	3/3	3/3	3/3	18/18	3/3
*Δbbi42Δbbq03Δbbk53*								
4-week PI	3/3	3/3	3/3	3/3	3/3	3/3	18/18	3/3

^*a*^ Node pairs were collected and cultured separately, all node pairs cultured consistently and are summarized here.

## DISCUSSION

Significant protein homology in *B. burgdorferi* has complicated the characterization of its many paralogous gene families. While individual PFam members have been evaluated, very few studies have produced a strain deficient in all members of a paralogous family. PFam53 consists of two operonic surface proteins (OspA/B) which were deleted using a polar insertion in OspA (72). PFam37 consists of five peptide binding proteins (OppAs) which are encoded on a chromosomal operon and among plasmids; the Opp system was abrogated by conditional deletion of a singular heterodimeric ATPase (OppDF) which drives the transport system (38). PFam52 presented an enticing focus for a multi-targeted mutagenesis approach given their high levels of homology, the minimal number of potentially functional paralogs, and their distribution among plasmids. In addition, PFam52 members were suspected of RpoS-regulated outer surface proteins, potentially indicating a role during infection.

AlphaFold models of the PFam52 members demonstrated a highly similar overall structure, with the greatest variation in the C-terminal region. While BBI42 and BBK53 have highly similar disordered regions at the C-terminus, BBQ03 extends the terminal alpha helix. This drastic change in the C-terminal structure may point to functional differences between I/K and Q proteins. To characterize these proteins *in vitro* and evaluate their phenotype during infection, we generated strains expressing only a single PFam52 member (double mutants) and a PFam52-deficient strain (*Δbbi42Δbbk53Δbbq03* triple mutant) for analysis. *bbi42*, *bbk53*, and *bbq03* each were demonstrated to be expressed in strain B31. Mapping of the transcription start sites (TSSs) confirmed that all three transcripts originate from a common start site and are expressed from identical RpoS-dependent promoters consistent with the consensus previously predicted by Caimano et al. (52) and all three proteins were shown to be RpoS-regulated. BBI42, BBK53, and BBQ03 were confirmed to be present on the outer surface of the spirochete, confirming the proteins’ potential to interact with the vector and/or host and were demonstrated to be readily detectable antigens during long-term infections. As noted above, sequence homology between the three PFam52 members required the use of single paralog expressing strains to assess surface localization and *rpoS*-dependent regulation of the individual paralogs. The use of single paralog-expressing strains is a limitation of this study as co-expression of the three PFam52 paralogs in wild-type *B. burgdorferi* could potentially influence their individual surface localization and/or regulation.

Surprisingly, the PFam52-deficient strain was able to colonize and disseminate in mice injected *via* needle inoculation regardless of dosage and over varying timepoints post-infection. *Δbbi42Δbbk53Δbbq03* was able to disseminate to all tissues, colonize tissues at wild-type levels, and persist for up to 10 weeks post-infection. Furthermore, tick studies demonstrated *Δbbi42Δbbk53Δbbq03* could be acquired by larvae and colonize the midgut at levels comparable to wild-type B31 throughout the molt to the nymphal stage. During transmission studies, the PFam52-deficient mutant also replicated to wild-type levels in the feeding nymph and transmitted to naïve mice and, again, disseminated at wild-type levels. These data confirm that the PFam52 paralogs are dispensable for *B. burgdorferi*’s complicated life cycle.

Though the mutant was assessed at each stage of the enzootic cycle, it is possible that our assessment of infectivity as demonstrated by dissemination and colonization of mouse tissues is overly simplistic. *B. burgdorferi* is known to encode proteins with highly redundant functions related to surface proteins that promote host interactions (4). For example, five proteins have been identified that bind fibronectin (BBK32, RevA/B, BB0347, and CspA) and many proteins have been shown to bind multiple host targets such as CspA which binds fibronectin, laminin, collagen, and plasminogen. Given these trends, it is possible that the phenotype of the PFam52-deficient mutant is masked due to functional redundancy among other proteins. In addition, our infection model focuses on typical tissue colonization sites historically evaluated in the mouse model (skin, heart, and joint). However, recent studies of the murine neurological components of *B. burgdorferi* infections have shown that spirochetes can be found in cerebrospinal fluid and the meninges during infection (73). It is possible that the mutant may demonstrate a phenotype within some of these non-traditional niches that are not routinely evaluated during murine infections. A final facet of *B. burgdorferi* infectivity that is often ignored is its broad host range, including avian species. One possible role for multiple paralogs, specifically for outer surface proteins, is to extend the host range for the spirochete to encompass the tick host range. Host-tropism has recently been shown for CspA, a PFam54 member using comparisons between a mouse and quail infection model (74). While we did not elucidate a role for the PFam52 members within this study, it is possible that these proteins play a role in a previously underappreciated facet of infection.

## MATERIALS AND METHODS

### Bacterial strains and culture conditions

The strains and plasmids used in this study are described in Table S1. *Escherichia coli* strain TOP10F’ (Life Technologies, Carlsbad, CA) was used as a cloning host. *E. coli* C41(DE3) (Lucigen, Middleton, WI) was utilized for the expression of recombinant protein. Lysogeny broth (LB) medium was used for *E. coli* cultivation and transformants were selected using 100 µg/mL of ampicillin, 100 µg/mL of spectinomycin, 50 µg/mL of kanamycin, or 5 µg/mL of gentamicin. *B. burgdorferi* strain B31 clone 54A (B31) was used in all studies (58). Unless otherwise noted, *B. burgdorferi* was cultured at 37°C with 3% CO_2_ in Barbour-Stoenner-Kelley-II (BSK-II) medium (75). Antibiotics were added when appropriate (150 µg/mL streptomycin, 150 µg/mL kanamycin, or 50 µg/mL gentamicin). Preparation and transformation of electrocompetent *B. burgdorferi* were conducted as previously described by Yang et al*.* (72).

### Bioinformatics and protein modeling

PFam52 paralog sequences from B31 were aligned using MUSCLE and visualized in ESPript using the secondary structure prediction of BBI42 (76, 77). For phylogeny, sequences of PFam52 members were collected from representative *Borrelia* strains, and multiple-sequence alignments were generated using MUSCLE and displayed with ESPript 3.0. The PHYLIP output file was submitted to PhyML for phylogenetic analysis and the Newick file generated was displayed using the Interactive Tree of Life (iTOL) (76–79). Protein modeling was performed with AlphaFold and visualized in Pymol (80). Electrostatics were calculated using the PDB2PQR/APBS software suite (81). RMSD values for aligned models were determined in ChimeraX 1.6.1 (82).

### Generation and confirmation of plasmids and strains used in this study

Oligonucleotides utilized in this study are listed in Table S2. Cloning fragments were PCR-amplified using TaKaRa PrimeSTAR HS DNA Polymerase (Clontech, Mountain View, CA, USA) and B31 genomic DNA (gDNA) as the template. Amplicons were gel purified, TA-cloned into pGEM-T Easy (Promega Corp., Madison, WI), and sequence confirmed. All *B. burgdorferi* transformants were screened for plasmid content.

#### PFam52 mutants

PFam52 deletion mutants were generated by directed allelic exchange using a sequential mutational approach. Mutagenesis constructs were generated using similar approaches. Flanking regions approximately 1 kb in size were generated for *bbi42* (accession no. NP_045573), *bbk53* (accession no. NP_045624), and *bbq03* (accession no. NP_051468). Primers introducing a 3′ AscI restriction site were used to amplify the upstream flanking region (5′ F1-BBI42 and 3′ F1-BBI42/K53-AscI for *bbi42*, 5′ F1-BBK53 and 3′ F1-BBI42/K53-AscI for *bbk53*, 5′ F1-BBQ03 and 3′ F1-BBQ03-AscI for *bbq03*). The downstream flanking region was amplified with a 5′ AscI and 3′ BssHII restriction site (5′ F2-BBI42/K53-AscI and 3′ F2-BBI42/K53-BssHII for *bbi42* and *bbk53*, 5′ F2-BBQ03-AscI and 3′ F2-BBQ03-BssHII). To ligate the two flanking regions, the downstream fragment was excised using BssHII and ligated into the vector containing the upstream fragment that was linearized with AscI. An antibiotic marker was ligated into the AscI restriction site located between the two fragments. For the sequential mutagenesis approach (Fig. 2A), three different antibiotic resistance markers were utilized; the P*flgB-aphI-*T7t Kan^r^ (83), the P*flgB-aadA-trpLterm* Strep^r^ (84), and P*flgB-aacC1-trpLterm* Gent^r^ (85) markers. These markers were provided by Dr. Scott Samuels (University of Montana). The latter two markers were modified using overlap extension PCR to mutate an intrinsic AscI site linking the gene to the *trpL* terminator. The final constructs contained an antibiotic marker that replaced an internal region of the *bbi42*, *bbk53*, or *bbq03* open reading frame (ORF) and designated pJSB528 (*bbi42::Kan*), pJSB532 (*bbk53::Kan*), pJSB569 (*bbk53::Gent*), pJSB592 (*bbk53::Strep*), and pJSB566 (*bbq03::Gent*).

To generate *Δbbk53Δbbq03*, the B31 parental clone was first transformed with pJSB532, and clones were selected with kanamycin. Interruption of *bbk53* was confirmed by PCR using primers that amplified from the Kan^r^ marker into the upstream flanking region of *bbk53* (5′ BBK53 Diag and 3′ Kan Diag). A confirmed *Δbbk53* clone was then electroporated with pJSB566 and transformants were selected with kanamycin and gentamycin. PCR with primers that amplified from the Gent^r^ marker into the upstream flanking region of *bbq03* (5′ BBQ03 Diag and 3′ Gent Diag) was used to confirm the integration of the Gent^r^ marker into *bbq03*.

To generate *Δbbi42Δbbq03*, B31 was electroporated with pJSB528, and transformants were selected with kanamycin. Interruption of *bbi42* was confirmed by PCR using primers that amplified from the Kan^r^ marker into the upstream flanking region of *bbi42* (5′ BBI42 Diag and 3′ Kan Diag). A single *Δbbi42* clone was transformed with pJSB566, and clones were selected with kanamycin and gentamycin. Integration of the Gent^r^ marker into *bbq03* was confirmed by PCR using primers that amplified from the Gent^r^ marker into the upstream flanking region of *bbq03* (5′ BBQ03 and 3′ Gent Diag).

To generate *Δbbi42Δbbk53*, *Δbbi42* was electroporated with pJSB569, and transformants were selected with kanamycin and gentamycin. A diagnostic PCR with primers that amplified from the Gent^r^ marker into the upstream flanking region of *bbk53* (5′ BBK53 Diag and 3′ Gent Diag) was used to confirm the interruption of *bbk53* by the Gent^r^ marker.

To generate the *Δbbi42Δbbk53Δbbq03* triple mutant, pJSB592 was electroporated into the *Δbbi42Δbbq03* clone. Transformants were selected with kanamycin, gentamycin, and streptomycin, and two *Δbbi42Δbbk53Δbbq03* clones were characterized. Interruption of *bbk53* was confirmed by PCR with primers that amplified from the Strep^r^ marker into the upstream flanking region of *bbk53* (5′ BBK53 and 3′ Strep Diag). Due to the high level of homology among *bbi42*, *bbk53*, and *bbq03* nucleotide sequences, a second set of confirmatory PCRs were conducted on *Δbbk53Δbbq03*, *Δbbi42Δbbq03*, *Δbbi42Δbbk53*, and *Δbbi42Δbbk53Δbbq03* clones. Primers were used that amplified one of two separate regions internal to the deleted section of the targeted ORFs (5′ IQK US Diag and 3′ IQK US Diag, 5′ IQK DS Diag and 3′ IQK DS Diag). The amplified regions include nucleotide polymorphisms that are gene-specific; thus, purified amplicons from *Δbbk53Δbbq03*, *Δbbi42Δbbq03*, and *Δbbi42Δbbk53* were sequence confirmed.

#### rpoS and irpoS strains

Generation of the B31 *ΔrpoS* mutant was described by Caimano et al. (21). To generate *B. burgdorferi* strains carrying an isopropyl-1-thio-β-D-galactopyranoside (IPTG)-inducible *rpoS*, designated *irpoS*, the pJSB104 inducible expression construct (86) was modified as follows. First, the P*flgB-aadA* Strep^r^ marker was replaced with the *aph*[3′]-*IIIa* Kan^r^ gene from the pJD44 shuttle vector (86), a critical step to remove the NdeI site within the Strep^r^ marker in pJSB104. Second, the *rpoS* ORF was amplified from strain 297 using primers that introduced NdeI and HindIII restriction enzyme sites at the 5′ and 3′ ends, respectively (5′ *rpoS* ORF-NdeI and 3′ *rpoS* ORF-HindIII). The amino acid sequences for the RpoS ORF of strains 297 and B31 are identical. The Bb*luc* + ORF was then replaced with the *rpoS* ORF to generate pJSB267. The inducible expression cassette was excised from pJSB267 with XbaI/HindIII and ligated into the Strep^r^ shuttle vector pJSB201 where the NdeI site was removed from Strep^r^ (87) and digested with the same enzymes. This construct, designated pJSB510, was electroporated into *Δbbk53Δbbq03*, *Δbbi42Δbbq03*, and *Δbbi42Δbbk53.* Transformants were selected with kanamycin, gentamycin, and streptomycin, and then confirmed to contain pJSB510 by plasmid recovery, restriction digestion, and sequencing. Resulting clones were designated *irpoS-Δbbk53Δbbq03*, *irpoS-Δbbi42Δbbq03*, and *irpoS-Δbbi42Δbbk53*.

### Cloning for recombinant proteins

Recombinant BBI42, BBK53, BBQ03, and OspC proteins were generated from B31 by PCR amplification with primers incorporated a 5′ BamHI and a 3′ EcoRI restriction site (5′ BBI42/K53 ORF-SP-BamHI and 3′ BBI42 ORF-HindIII for BBI42, 5′ BBI42/K53 ORF-SP-BamHI and 3′ BBK53 ORF-HindIII for BBK53, 5′ BBQ03 ORF-SP-BamHI and 3′ BBQ03 ORF-HindIII for BBQ03, 5′ B31 OspC ORF-SP-BamHI and 3′ B31 OspC ORF-SP-EcoRI for OspC). LipoP 1.0 predicted a signal peptidase II cleavage site (67) for the PFam52 ORFs and primers excluded the N-terminal 16 (BBI42), 17 (BBK53 and BBQ03) amino acids of each paralog (Fig. 1C), and 18 amino acids for OspC. The ORF fragments were sequence confirmed and ligated into the pPROEX-HTb expression vector (Life Technologies) to generate pJSB672 (*bbi42*), pJSB673 (*bbk53*), pJSB469 (*bbq03*), and pJSB678 (*ospC*).

### Expression and purification of recombinant protein

pProEX-HTb generates recombinant protein tagged with an N-terminal His_6_ and a tobacco etch virus (TEV) protease cleavage site in the linker region. To produce recombinant protein, pJSB672, pJSB673, pJSB469, and pJSB678 were transformed into *E. coli* C41(DE3) and cultures were induced with 1 mM IPTG for 3 h. Recombinant His_6_-tagged recombinant PFam52 proteins were affinity-purified as an insoluble protein as previously described (87).

### Generation of BBQ03 antisera

Three- to four-week-old Sprague-Dawley rats were purchased from Harlan (Indianapolis, IN) and experiments were performed at UAMS under the aforementioned approved animal use protocol. Antisera were generated in rats as previously described (87). In brief, 25 µg of recombinant BBQ03 was combined 1:1 with complete Freund’s adjuvant (Sigma, St. Louis, MO), emulsified, and used to immunize rats intraperitoneally. Rats were boosted twice every 4 weeks as described above with incomplete Freund’s adjuvant (Sigma) and serum was collected 2 weeks after final immunization.

### Immunoblot analyses

Immunoblot analyses were performed as previously described. In brief, cell lysates equivalent to 2 × 10^7^ bacteria were loaded in each lane of a 12.5% SDS-PAGE gel and separated by electrophoresis. Proteins were transferred to nitrocellulose membranes for colorimetric detection using 4-chloro-1-napthol as the substrate or for chemiluminescence detection with SuperSignal West Dura Extended Duration Substrate (Thermo Scientific). Detection of FlaB and OspC was facilitated by antisera and antibodies previously described (45, 88). BBQ03-specific antisera production is described above. Quality issues with the RpoS-specific monoclonal antibody led us to use OspC levels as a surrogate to confirm RpoS production. All Blue Precision Plus protein marker (Bio-Rad Laboratories, Hercules, CA) was used as the molecular mass standard for immunoblots.

### Cultivation of *B. burgdorferi* for expression analyses

To assess the impact of endogenous *rpoS* activation, cultures were inoculated at a starting density of 10^3^ bacteria/mL in BSK-II media containing appropriate selection and grown for 7 days at 37°C/pH 6.8 or at a starting density of 10^4^ bacteria/mL and grown for 20 days at room temperature/pH 7.5. For IPTG-dependent induction of *rpoS*, *irpoS* strains were inoculated at a starting density of 10^3^ bacteria/mL in BSK-II and grown at 37°C/pH 7.5. Cultures were split when bacteria reached the early-logarithmic growth and 0.1 mM IPTG was added to one aliquot while the other was left untreated and grown for 12 h. Protein samples were prepared as described below.

### TSS mapping

Total RNA was isolated from *in vitro* cultivated bacteria using TRIzol reagent (Life Technologies) according to the manufacturer’s protocol. RNA was purified and DNase treated using the RNeasy minikit and RNase-free DNase I, respectively (Qiagen Inc., Valencia, CA). The presence of contaminating DNA was tested for amplification using TaKaRa EmeraldAmp GT PCR master mix (Clontech) and primers specific for *flaB*. For TSS identification of the PFam52 paralogs, single paralog expressing strains were subjected to 5′ Rapid Amplification of cDNA Ends (RACE) using 5′ RACE system v2.0 (Life Technologies). PFam52-specific primers 5′ RACE-GSP1 and 5′ RACE-GSP2 were used for the generation of cDNA and fragment PCR amplification, respectively. Gel-purified fragments were cloned into pGEM-T Easy and multiple clones were sequenced using vector-specific primers to identify the TSS.

### Cell surface localization studies

To confirm the localization of the PFam52 paralogs to the cell surface, proteinase K accessibility assays were performed as previously described (89). In brief, 2 × 10^9^ spirochetes were resuspended in PBS with 5 mM MgCl_2_ (PBS-MgCl_2_) in the absence or presence of 400 µg/mL proteinase K (Life Technologies) and incubated at room temperature for 60 minutes. 5 mM phenylmethylsulfonyl fluoride (Thermo Scientific, Rockland, IL) was used to stop the reaction. Samples were then prepared for SDS-PAGE and immunoblot analysis for PFam52 paralogs, FlaB (periplasmic control), and OspC (outer membrane control).

### Routine infection studies

Five- to six-week-old female C3H/HeNCrl mice or C3H/HeJ mice were purchased from Charles River Laboratories (Raleigh, NC) and Jackson Laboratories (Bar Harbor, ME) and experiments were performed at UAMS and UConn under the aforementioned approved animal use protocols. Clones were grown with appropriate antibiotic selection to the mid-log growth phase, and cultures were enumerated by dark-field microscopy and appropriately diluted prior to injection. Mice were infected by intradermal needle inoculation in the sternal or dorsal region as previously described (90). For 50% infectious dose (ID_50_) experiments, mice were infected with serial 10-fold dilutions containing doses of 10^2^ to 10^5^ bacteria. At 2 weeks post-infection, tissue samples (e.g., ear punch, distal skin, heart, tibiotarsal, bladder, and paired primary axillary, accessory axillary, subiliac, or popliteal nodes) were collected and cultured in BSK-II medium containing *Borrelia* antibiotic mixture (Monserate, San Diego, CA). In addition, heart, tibiotarsal, and skin samples were collected and preserved for qPCR. Heart and knee tissues were collected for histopathology assessment. Cultures containing tissue samples were examined by dark-field microscopy for the presence of spirochetes up to 4 weeks after collection. For chronic infection experiments, mice were infected with a single dose of 10^4^ bacteria. Tissues were collected at 2, 6, and 10 weeks post-infection and assessed for infection as described above for the ID_50_ experiments.

### Larval acquisition and generation of fed nymphs

Mice were needle-inoculated with 10^4^ bacteria and infection was confirmed at 2 weeks post-infection by positive ear punch cultures. Infected mice were used as a blood meal source for 200–300 pathogen-free *I. scapularis* larvae (Oklahoma State University, Stillwater, Oklahoma). Ten replete larvae were collected from each mouse and evaluated for spirochete burdens by qPCR (91, 92) and semisolid plating as previously described (39, 93). The remaining fed larvae were allowed to molt to nymphs over super-saturated potassium sulfate in an environmental incubator. After molt, 10 flat nymphs per mouse were collected for qPCR and semisolid plating. Finally, 10 flat nymphs were fed to repletion *via* the capsule feeding method (94) on three mice/strains and collected for qPCR and semisolid plating. Mice were evaluated for infection at 2 weeks post-infection as described above.

### Histological assessment

Heart and knee tissues for histological assessment were processed, and sagittal sections were scored blindly for the presence and severity of arthritis and carditis as previously described (87). Individual scores were totaled for maximum possible scores of 7 or 13 for carditis and arthritis, respectively.

### qPCR analyses of spirochete burdens

For needle-inoculation studies, DNA was extracted from infected tissues with the High Pure PCR Template Preparation Kit (Roche Applied Sciences) according to the manufacturer’s instructions, and collagenase was added during cell lysis. Copies of *flaB* and the murine β-actin gene were determined using the TaqMan Fast Advanced Master Mix (Life Technologies); primer and probe sets were described in Groshong et al. (87). Spirochetal burdens are reported as copies of *flaB* per 10^6^ copies of mouse β-actin. For tick studies, DNA was extracted from infected tissues with DNeasy Blood and Tissue Kit (Qiagen) with collagenase added during cell lysis. Copies of *flaB* and murine nidogen were determined using iQ Supermix and primer and probe sets as described in Pal et al. and Tupin et al., respectively (92, 95). Spirochetal burdens are reported as copies *flaB* per 10^6^ copies of mouse nidogen. DNA was extracted from pools of 10 ticks using the Puregene Yeast and Bacteria Kit (Qiagen) and 1:10 dilutions were assayed for *flaB* using Q Supermix and primer and probe sets as described in Pal et al. (92)

### Statistical analyses

The ID_50_ for B31 and *Δbbi42Δbbk53Δbbq03* was calculated from the mouse infections as previously described (87, 96). Comparisons between ID_50_ values were made using a generalized linear model with a probit link function. All calculations for ID_50_ were carried out using R version 3.0.1 software (R Foundation for Statistical Computing, Vienna, Austria). Burden analyses were compared using *t-*test.

## SUPPLEMENTAL MATERIAL

Supplemental material iai.00214-24-s0001.pdf Tables S1 and S2; Fig. S1 to S5.

ASM does not own the copyrights to Supplemental Material that may be linked to, or accessed through, an article. The authors have granted ASM a non-exclusive, world-wide license to publish the Supplemental Material files. Please contact the corresponding author directly for reuse.
