# Supplementary material for: The plasmid-encoded members of paralogous gene family 52 are dispensable to the enzootic cycle of Borrelia burgdorferi
Source: Infect Immun. 2024 Aug 9;92(10):e00214-24. doi: 10.1128/iai.00214-24 (PMC11475691; doi:10.1128/iai.00214-24)
Supplement: Supplemental material — Tables S1 and S2; Fig. S1 to S5. [file iai.00214-24-s0001.pdf]

Table S1. Plasmids and strains used in this study.

Plasmid or strain	Description[a]	Source
Plasmids		
pGEM-T Easy	TA-cloning vector; Amp^r	Promega
pProEX-HTb	Expression construct, N-terminal and TEV-cleavable His$_6$; Amp^r	Life Technologies
pJSB528	pGEM-T Easy-*bbi42*::P*flgB-aphI* mutation construct; Kan^r	This study
pJSB532	pGEM-T Easy-*bbk53*::P*flgB-aphI* mutation construct; Kan^r	This study
pJSB569	pGEM-T Easy-*bbk53*::P*flgB-aacC1* mutation construct; Gent^r	This study
pJSB592	pGEM-T Easy-*bbk53*::P*flgB-aadA* mutation construct; Spec^r Strep^r	This study
pJSB566	pGEM-T Easy-*bbq03*::P*flgB-aacC1* mutation construct; Gent^r	This study
pJSB634	pGEM-T Easy-*rpoS*::P*flgB-aadA* mutation construct; Spec^r Strep^r	(1)
pJSB104	Shuttle vector with IPTG-inducible Bb*luc*+ luciferase; Spec Strep^r	(2)
pJD44	pJD7-based shuttle vector with *aph*[3′]-*IIIa*; Kan^r	(2)
pJSB267	Shuttle vector with IPTG-inducible *rpoS*; Kan^r	This study
pJSB201	pJD7 with the NdeI site in P*flgB-aadA* mutated; Spec^r Strep^r	(3)
pJSB510	Derivative of pJSB267 with P*flgB-aadA*; Spec^r Strep^r	This study
pJSB82	pJD7::P*flaB*-Bb*luc*+; Spec^r Strep^r	(2)
pJSB672	pProEX-HTb::*bbi42* ORF, no signal peptide; Amp^r	This study
pJSB673	pProEX-HTb::*bbk53* ORF, no signal peptide; Amp^r	This study
pJSB469	pProEX-HTb::*bbq03* ORF, no signal peptide; Amp^r	This study
pJSB678	pProEX-HTb::*ospC* ORF, no signal peptide; Amp^r	This study
**Strains**		
E. coli		
TOP10F'	F'[*lacI*q, Tn*10*(Tet^r)] *mcrA* Δ(*mrr-hsd*RMS-*mcr*BC) ϕ80*lacZ*ΔM15 Δ*lac*X74 *rec*A1 *ara*Δ139 Δ(*ara-leu*)7697 *gal*U *gal*K *rps*L (Str^r) *end*A1 *nup*G	Life Technologies
C41(DE3)	F$^-$ *ompT hsdSB* (r_B- m_B-) *gal dcm* (DE3)	Lucigen
B. burgdorferi		
B31	Clone 5A4, isolated from low passage B31 and contains all plasmids	(4)
ΔrpoS	B31 transformed with pJSB634; Strep^r	(1)
Δbbk53	B31 transformed with pJSB532; Kan^r	This study
Δbbk53Δbbq03	*Δbbk53* transformed with pJSB566; Kan^r Gent^r	This study
Δbbi42	B31 transformed with pJSB528; Kan^r	This study
Δbbi42Δbbk53	*Δbbi42* transformed with pJSB569; Kan^r Gent^r	This study
Δbbi42Δbbq03	*Δbbi42* transformed with pJSB566; Kan^r Gent^r	This study
Δbbi42Δbbk53Δbbq03	*Δbbi42Δbbq03* transformed with pJSB592; Kan^r Gent^r Strep^r	This study
irpoS-Δbbk53Δbbq03	*Δbbk53Δbbq03* transformed with pJSB510; Kan^r Gent^r Strep^r	This study
irpoS-Δbbi42Δbbk53	*Δbbi42Δbbk53* transformed with pJSB510; Kan^r Gent^r Strep^r	This study
irpoS-Δbbi42Δbbq03	*Δbbi42Δbbq03* transformed with pJSB510; Kan^r Gent^r Strep^r	This study

[a]Spec, spectinomycin; Strep, streptomycin; Amp, ampicillin; Kan, kanamycin; Gent, gentamycin.

References

1. Caimano, M.J., et al., The RpoS gatekeeper in Borrelia burgdorferi: an invariant regulatory scheme that promotes spirochete persistence in reservoir hosts and niche diversity. Front Microbiol, 2019. 10: p. 1923, doi:10.3389/fmicb.2019.01923.
2. Blevins JS, Hagman KE, Norgard MV. 2008. Assessment of decorin-binding protein A to the infectivity of Borrelia burgdorferi in the murine models of needle and tick infection. BMC Microbiol 8:82, doi:10.1186/1471-2180-8-82.
3. Groshong AM, Gibbons NE, Yang XF, Blevins JS. 2012. Rrp2, a prokaryotic enhancer-like binding protein, is essential for viability of Borrelia burgdorferi. J Bacteriol 194:3336-42, doi:10.1128/JB.00253-12.
4. Purser JE, Norris SJ. 2000. Correlation between plasmid content and infectivity in Borrelia burgdorferi. Proc Natl Acad Sci U S A 97:13865-70, doi:10.1073/pnas.97.25.13865.

TABLE S2. Oligonucleotide primers used in this study

Primer designation	Sequence[a]
5' F1-BBI42	GGGTTTAATCTAAGGATCAAGATGAGGAATTTAG
3' F1-BBI42/K53-AscI	GGCGCGCCTCATATATGCTCCTTACCTACC
5' F2-BBI42/K53-AscI	GGCGCGCCTGAACATCGTTTATTGGTTGAG
3' F2-BBI42/K53-BssHII	GCGCGCAAATAAAACATTAAGTACAATAACCCAA
5' F1-BBK53	TTAATATAATTTCGTAGGTTTTATATTTATCGGTTTC
5' F1-BBQ03	GCTGTTCAACTGCAATTGTTGCACTT
3' F1-BBQ03-AscI	TGGCGCGCCTTGTTCCTGATTATCA
5' F2-BBQ03-AscI	AGGCGCGCCCTGAGTATGATCGTTT
3' F2-BBQ03-BssHII	AGCGCGCACAAGTTGAGATGCCGAA
5’ BBI42 Diag	GGTGGAGTATTCTTTGTTATTTTTAGA
5’ BBK53 Diag	GCACATTTTGTGTGAACTGCTATTTCTAT
5’ BBQ03 Diag	GTTATAGAAAAATCTATCTTGGTAGG
3’ Kan Diag	CGCTGAGCAATAACTAGCATAACC
3’ Gent Diag	GTTGGGCATACGGGAAGAAGTG
3’ Strep Diag	GCTAGACAGGCTTATCTTGGAC
5’ IQK US Diag	CATATATGAGGATTTTGGTTGGCG
3’ IQK US Diag	CAGTAACAATCTCATCGGAACCC
5’ IQK DS Diag	GGGTTCCGATGAGATTGTTACTG
3’ IQK DS Diag	AATACTCCTCATTATAGTCAC
5’ *rpoS* ORF-NdeI	CATATGAACATATTTAGTAATGAGGATTTAAAC
3’ *rpoS* ORF-HindIII	AAGCTTAATTTATTTCTTCTTTTAATTTTTTAAGAACTC
5’ BBI42/K53 ORF-SP-BamHI	GGATCCTATTTGCCTGATAATCAGGAACAAGCTG
3’ BBI42 ORF-HindIII	AAGCTTATGTAGGTAAAATAGGAACTGGTC
3’ BBK53 ORF-HindIII	AAGCTTATGTAGGTAAAATAGAAACTGGGC
5’ BBQ03 ORF-SP-BamHI	GGATCCTATTTGCCTGATAATCAGGAACAAGCTGTTC
3’ BBQ03 ORF-HindIII	AAGCTTTATCATAAAATTTTTCCATTAATTGTATTTC
5’ BBQ03 full ORF-NdeI	CATATGAGGATTTTGGTTGGCGTTTTCATAATAGC
5’ I/K full ORF-NdeI	CATATGAGGATTTTGGTTGGCGTTTGTATAATAGC
5’ B31 OspC ORF-SP-BamHI	GGATCCAAAGATGGGAATACATCTGCAAATTCTGCTG
3’ B31 OspC ORF-SP-EcoRI	GAATTCTTAAGGGTTTTTTGGACTTTCTGCCAC
5’ RACE-GSP1	CTCCTCATTATAGTCACTCACTGG
5’ RACE-GSP2	CTCAACCAATAAACGATGTTCAGA
FlaB-ABI-F	TTATGCAGCTAATGTTGCAAATCTT
FlaB-ABI-R	TTCCTGTTGAACACCCTCTTGA
FlaB-ABI-Probe-FAM	CTCAAACTGCTCAGGCTGCACCGG
Act-ABI-F	GACGGACTACCTCATGAAGATCCT
Act-ABI-R	CACGCACGATTACCCTCTCA
Act-ABI-Probe-MAX	ACCGAGCGTGGCTACAGCTTCATCA
FlaB-F	CTTTTCTCTGGTGAGGGAGCTC
FlaB-R	GCTCCTTCCTGTTGAACACCC
FlaB-Probe-FAM	CTTGAACCGGTGCAGCCTGAGCA
Nido-F	CCCCAGCCACAGAATACCAT
Nido-R	AAAGGCGCTACTGAGCCGA
Nido-Probe-FAM	CCGGAACCTTCCCACCCAGC

[a] Relevant restriction sites are underlined.

```
                          1        10         20         30         40         50
B. burgdorferi B31 BBI42  BBIMRILVGVCII.ALALLGCYLPDNQEQAVQTFFEN.S.ESSDMGSDEIVTE.....GI
B. burgdorferi B31 BBK53  BBKMRILVGVCIIAALALLGCYLPDNQEQAVQTFFEN.S.ESSDMGSDEIVTE.....GI
B. burgdorferi B31 BBQ03  BBQMRILVGVFIIAALALLGCYLPDNQEQAVQTFFEN.S.ESIDMGSDEIVTE.....GI
B. burgdorferi JD1 0912   MRILVGVCII.ALALLGCYLPDNQEQAVQTFFEN.S.ESSDMGSDEIVTE.....GIFSS
B. burgdorferi JD1 BBE04  BBEMRILVGVCII.ALALLGCYLSDNQEQAVQTFFEN.S.ESSDMGSDEIVTE.....GI
B. burgdorferi JD1 BBH47  BBHMRILVGVCII.ALALLGCYLPDNQEQAVQTFFEN.S.ESSDMGSDEIVTE.....GI
B. burgdorferi JD1 BBK37  BBKMRILVGVCIIAALALLGCYLPDNQEQAVKTFFEN.SEESSDMGSDEIVTE.....GI
B. mayonii 05655          MRILVGVCII.ALALLGCYLFDKKGQAAQTFFEN.S.ESSDMGSDEIVTE.....GIFSS
B. afzelii J0038          JMKTLVGACIV.TLAVLGCYLPDKQKQAVQTFFKN.S.QSSDIGSDEIVADEIVADGIFD
B. afzelii A0001          AMKTLVGACIV.TLAVLGCYLPDKQKQAVQTFFKN.S.QSSDIGSDEIVADEIVADGIFD
B. afzelii AA0001         AAMRILVGVCII.ILALLGCYLPDKQEQAVQAFFDENSPQSSDMGSNEIVTE.....GIF
B. afzelii G0020          GMRILVGVYII.TLALFACYLPNKQEQSVQTFFENLEMESYDKGSDEIITK.....GIFA
```

```
                                60        70        80        90       100       110
B. burgdorferi B31 BBI42  FSSBBILKLYASEHRLLVEIKKTLISLKDPN.YRGVVLPV.SDYNEEYFNKFFLDLGSEQ
B. burgdorferi B31 BBK53  FSSBBKLKLYASEHRLLVEIKKTLISLKDPN.YRDVVRPV.SDYNEEYFNKFFLDLGSEQ
B. burgdorferi B31 BBQ03  FSSBBQLKLYASEHRLLVEIKKTLISLKDPN.YRD.VPPV.RDYNEEYFNKFFLDLGSEQ
B. burgdorferi JD1 0912   ...LKLYASEHRLLVEIKKTLISLKDPN.YRGVVRPV.SDYNEEYFNKFFLDLGSEQSKD
B. burgdorferi JD1 BBE04  FSSBBELKLYASEHRLLVEIKKTLISLKDPN.YRD.VPPV.RDYNEEYFNKFFLDLGSEQ
B. burgdorferi JD1 BBH47  FSSBBHLKLYASEHRLLVEIKKTLISLKDPN.YRDVVRPV.SDYNEEYFNKFFLDLGSEQ
B. burgdorferi JD1 BBK37  FSSBBKLKLYASEHRLLVEIKKTLISLKDPN.YRD.VPPV.RDYNEEYFNKFFLDLGSER
B. mayonii 05655          ...LKLYASEHRLLVEIKKTLYTLQGLE.SRE.FPPL.PDYNEEYFNKFFLDLGSERSKD
B. afzelii J0038          N..JLKLYMTEHNLLVDIKKTIISLKDPN.YRA.VPPV.RDYNEEYFNKFFLDLGSERSK
B. afzelii A0001          N..ALKLYMTEHNLLVDIKKTIISLKDPN.YRA.VPPV.RDYNEEYFNKFFLDLGSERSK
B. afzelii AA0001         AN.AALKLYVAEHRLLIDIKETLISLKDPN.YRN.VPPVGKGYNEENFNKFFLDLGSERS
B. afzelii G0020          N..GLKLYIAEHRLLVDIKKTLDGLKYGSGGRD.VPPV.SDYNEEYFNKFFLDLGSERSK
```

```
                                    120       130       140       150       160
B. burgdorferi B31 BBI42  SKDLBBILFIMVKNEQNNNKFMRIVRWLYSCIEELYSPDIKYSGEEGSPEYYRN....MP
B. burgdorferi B31 BBK53  SKDLBBKLFIMVKNEQNNNKFMRIVRWLYSCIEELYSLDIKYSGE.GSHEYNRN....MP
B. burgdorferi B31 BBQ03  SKDLBBQLFGRVKNEQ.NNKFKSEVYWLYSCISELYSPDIKYSGEEGSPEYDRF....MP
B. burgdorferi JD1 0912   LIK.LFIMVKNEQNNNKFMRIVRWLYSCIEELYSPDIKYSGEEGSPEY............
B. burgdorferi JD1 BBE04  SKDLBBELFIMVKNEQNNNKFMREVHWLYSCIEELYSPDIKYSGEEGV.EYVRD....MP
B. burgdorferi JD1 BBH47  SKDLBBHLFIMVKNEQNNNKFMRIVRWLYSCIEELYSLDIKYSGE.GSHEYNRN....MP
B. burgdorferi JD1 BBK37  SKDLBBKLFGRVKNEQ.NNKFKSEVYWLYSCISELYSLDIKYSGEEGSPEYDRF....MP
B. mayonii 05655          LIK.LFGRVKNEQ.NNKFKSEVYWLYLCIRDLYSPDIKYSGEKGSPEYDRF....MP.RP
B. afzelii J0038          ELIKJLFGKIKNEQNDDKFKREAYWLYSCIRDLYSSDIKYSGENGN.EYIVD....MPRR
B. afzelii A0001          ELIKALFGKIKNEQNDDKFKREAYWLYSCIRDLYSSDIKYSGENGN.EYIVD....MPRR
B. afzelii AA0001         KELIAALFGRIKNDR..NLFEKKVSLLFYCIEDNSSNGIDFRSINAI..FDEE.......
B. afzelii G0020          ELIKGLFGIIKNDR.NDRFKHAVHWLYSCIRELYSPDIKYSGESGH.EYYSNGSEVVILR
```

```
                             170       180
B. burgdorferi B31 BBI42  .RPTABBIQYLKVKRYDYNRPVPILPT
B. burgdorferi B31 BBK53  .RPTABBKQYLKVKRYDYNSPVSILPT
B. burgdorferi B31 BBQ03  .RPTABBQQYLKVKREIERNTINGKIL
B. burgdorferi JD1 0912   ....................
B. burgdorferi JD1 BBE04  RRPTABBEQYLKVKAYF.....
B. burgdorferi JD1 BBH47  .RPTABBHQYLKVKRYDYNSPVSILPT
B. burgdorferi JD1 BBK37  .RPTABBKQYLKVKREIERNTINGKIL
B. mayonii 05655          TAYQ.QYLKVKREIERNTFNMVVLY
B. afzelii J0038          PTIDQJQYLKVKGVIEQYALR.
B. afzelii A0001          PTIDQAQYLKVKGVIEQYALR.
B. afzelii AA0001         .....AA....EKGVIK.....
B. afzelii G0020          PTIDEGQYLKVKKVIEHTHTLK
```

Fig. S1. Alignment of PFam52 paralogs from different Lyme *Borrelia* species. Red highlight denotes identity and blue box denotes similarity.

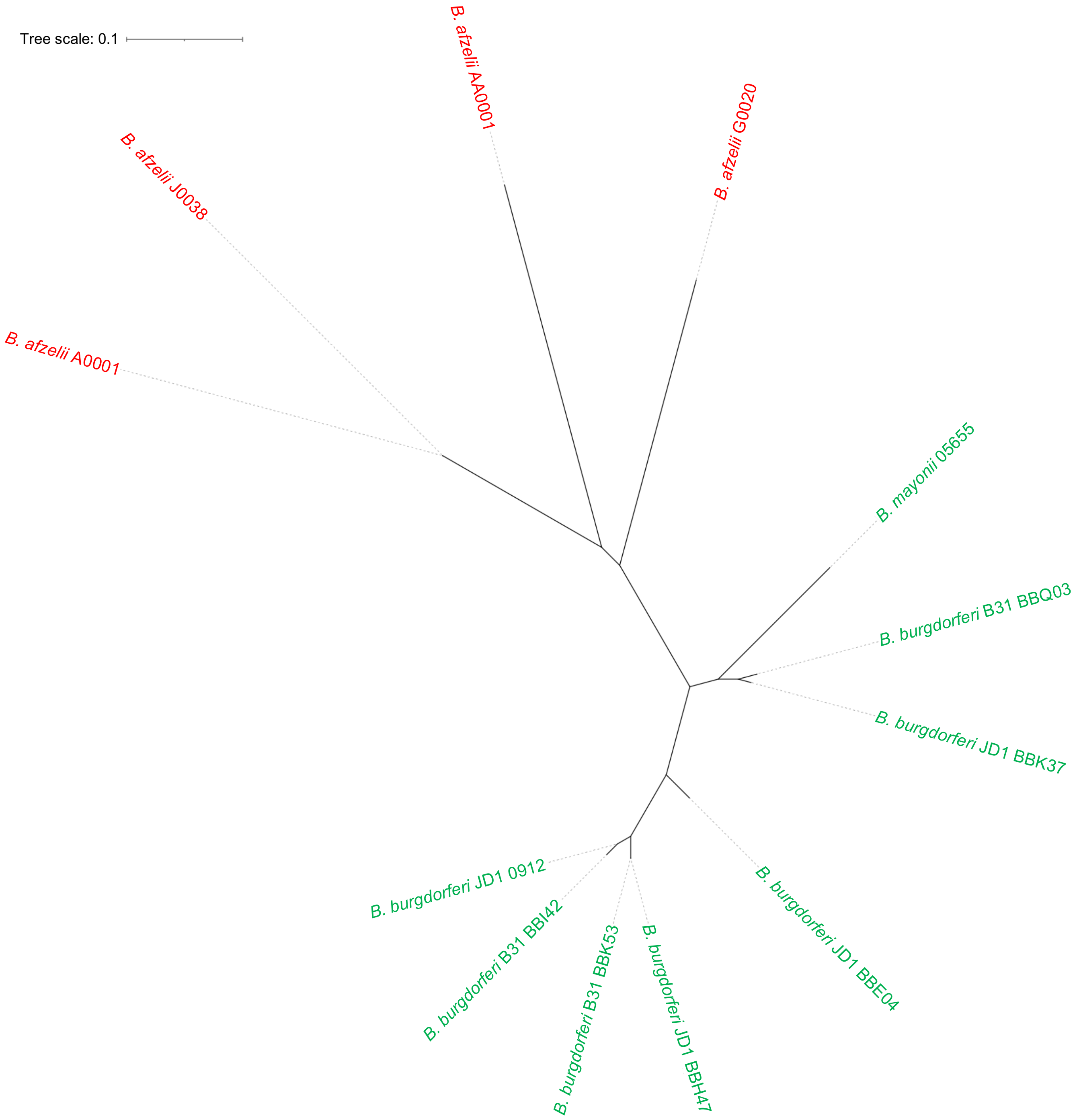

Fig. S2. Unrooted phylogenetic tree of PFam52 paralogs in different Lyme *Borrelia* species based off the alignment of Fig. S1. Red labels represent Old World *Borrelia* and green labels represent New World *Borrelia*.

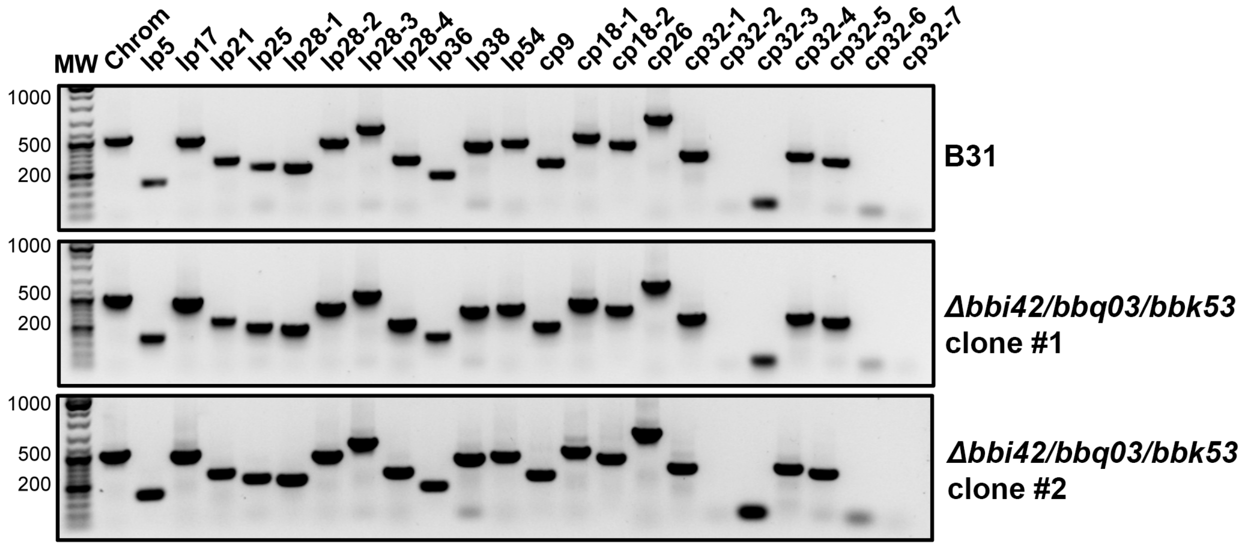

Fig. S3. Plasmid profile of strains used for murine infections. The presence of endogenous plasmids for strain B31 and two *Δbbi42Δbbk53Δbbq03* clones were assayed by PCR. Relevant molecular weight standards (MW) are designated to the left in basepair and plasmid designations are above.

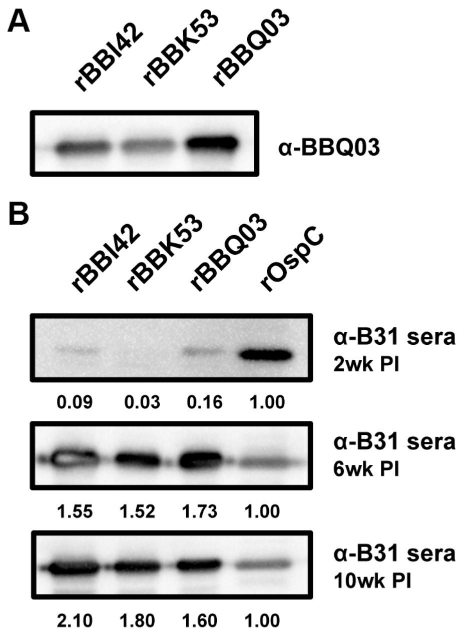

Fig. S4. (A) BBQ03 specific antiserum recognition of PFam52 paralogs. Immunoblot using BBQ03 antiserum blotted against recombinant BBI42, BBK53, and BBQ03 protein from *E. coli* lysates. Equivalent concentrations of recombinant BBI42, BBK53, and BBQ03 were determined by Sypro stain, and protein lysates were blotted with BBQ03 antiserum to assess cross-reactivity against all PFam52 paralogs. (B) Assessment of PFam52 paralog antibody response during *B. burgdorferi* infection of mice. Sera from mice infected with 10^4 spirochetes of B31 at two-, six-, and ten-weeks post-infection (PI). Pooled infected sera were blotted against *E. coli* lysates expressing recombinant BBI42, BBK53, BBQ03, and OspC, and densitometry was used to quantify signal and measure levels relative to OspC at the same timepoint.

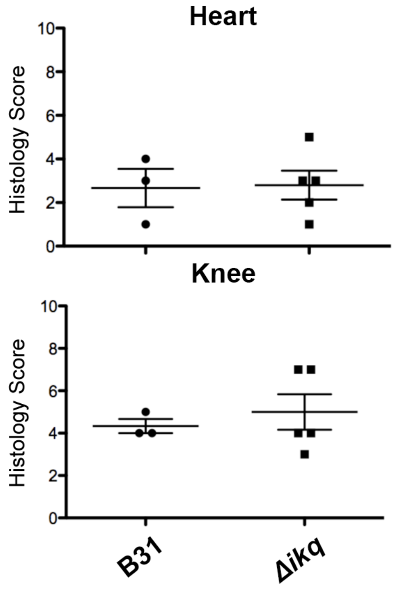

Fig. S5. Histopathological assessment of B31 and *Δbbi42Δbbk53Δbbq03* (*Δikq*) infected heart (top panel) and knee (lower panel) from mice infected with 10^3 spirochetes at two-weeks PI. Error bars represent SEM and differences between wild-type mutant were not statistically significant.
